# Tofacitinib, an oral Janus kinase inhibitor, in patients from Brazil with rheumatoid arthritis: Pooled efficacy and safety analyses

**DOI:** 10.1097/MD.0000000000011609

**Published:** 2018-08-03

**Authors:** Andrea B.V. Lomonte, Sebastião C. Radominski, Flora M.D. Marcolino, Claiton V. Brenol, Cristiano A.F. Zerbini, Erika G. García, Ermeg L. Akylbekova, Ricardo Rojo, Dario Ponce de Leon

**Affiliations:** a Centro Paulista de Investigação Clínica, São Paulo; b Universidade Federal do Paraná, Curitiba; c Faculdade de Medicina da Universidade de São Paulo, São Paulo; d Universidade Federal do Rio Grande do Sul, Hospital de Clínicas de Porto Alegre, Porto Alegre, Brazil; e Pfizer Inc, Collegeville, PA; f IQVIA, Durham, NC; g Pfizer Inc, Groton, CT; h Pfizer Inc, Lima, Peru.

**Keywords:** Brazil, efficacy, rheumatoid arthritis, safety, tofacitinib

## Abstract

**Background:**

Tofacitinib is an oral Janus kinase inhibitor for the treatment of rheumatoid arthritis (RA). Efficacy and safety of tofacitinib in Brazilian patients from Phase 2 (P2) and Phase 3 (P3) global studies of up to 24 months’ duration were evaluated.

**Methods:**

Data were pooled from Brazilian patients with RA and an inadequate response to conventional synthetic or biologic disease-modifying antirheumatic drugs enrolled in P2/P3 tofacitinib studies who received tofacitinib 5 or 10 mg twice daily (BID), or placebo, as monotherapy or in combination with methotrexate. Efficacy, safety, and patient-reported outcomes were assessed over 24 months.

**Results:**

Patients (226) from Brazil were treated in tofacitinib global P2/P3 studies. At Month 3, there were improvements in American College of Rheumatology 20/50/70 response rates, Disease Activity Score in 28 joints, erythrocyte sedimentation rate, and Health Assessment Questionnaire-Disability Index scores with both tofacitinib doses. Improvements from baseline in pain, fatigue, and health-related quality of life with tofacitinib 5 and 10 mg BID were reported. Efficacy improvements were sustained up to Month 24. The most frequent class of adverse events was infections and infestations. No cases of tuberculosis or other opportunistic infections were reported.

**Conclusion:**

In a Brazilian subpopulation of patients with RA, tofacitinib reduced disease signs and symptoms and improved physical function up to Month 24, with a safety profile consistent with findings from global studies.

## Abstract 2

Supplemental Digital Content is available in the text

## 1 Introduction

Rheumatoid arthritis (RA) is a chronic, progressive, systemic inflammatory disease that mainly affects the synovial membranes of joints, eventually resulting in bone and cartilage destruction.^[1]^ The estimated prevalence of RA in Brazil is 0.5%,^[2]^ although regional differences exist and prevalence ranges from 0.2% to 1.0% in South East and North Brazil, respectively.^[3]^

In Brazil, there may be barriers to optimal RA treatment, including inadequate access to patient care in the public health care system and medication costs in the private system.^[4]^ Moreover, the uneven distribution of rheumatologists and health care services across the different regions of Brazil and limited provision of specialized services in some regions may lead to referral delays and lack of appropriate treatment.^[3,5]^ Other challenging aspects for the management of patients with RA include endemic-epidemic transmissible diseases, which are still a public health concern in some regions of Brazil [e.g., tuberculosis (TB), dengue fever, visceral leishmaniasis],^[6]^ and may affect both the diagnosis and management of RA.^[5]^

Consensus guidelines developed by the Brazilian Society of Rheumatology (SBR) for the treatment for RA recommend conventional synthetic disease-modifying antirheumatic drugs [csDMARDs; particularly methotrexate (MTX)], as first-line treatment. For patients who fail to respond to 2 or more csDMARDs, biologic DMARDs [bDMARDs; mainly tumor necrosis factor inhibitors (TNFi)] are recommended.^[5]^ In Brazil, the bDMARDs infliximab, etanercept, adalimumab, golimumab, certolizumab, abatacept, rituximab, and tocilizumab are currently provided free of charge via the public health care system, in accordance with the Brazilian guidelines.^[5]^ However, in different regions of Brazil, the choice of bDMARD may vary depending on social, educational, and demographic factors, such as the lack of infusion centers for the administration of intravenous (IV) medication and difficulties experienced by some patients and their families with subcutaneous (SC) administration of treatment.^[5]^

Although bDMARDs have substantially improved the management of RA, globally 20% to 30% of bDMARD-treated patients still have active disease,^[7]^ and there remains an unmet need for alternative RA therapies that allow a greater proportion of patients to reach treatment goals than currently available agents.^[8]^ Furthermore, bDMARDs are limited by their IV or SC use, and orally available treatments are desirable. In respect of this, many patients with RA would prefer an orally administered treatment to an injectable therapy.^[9]^ To meet these unmet needs, orally administered small molecule compounds targeting intracellular signaling pathways have been developed, such as tofacitinib.

Tofacitinib is an oral Janus kinase (JAK) inhibitor for the treatment of RA.^[10]^ The clinical efficacy and safety of tofacitinib 5 mg twice daily (BID) and tofacitinib 10 mg BID have been reported in patients with RA in Phase 2 (P2),^[11–15]^ Phase 3 (P3),^[16–21]^ and long-term extension^[22,23]^ clinical trials. Tofacitinib 5 mg BID was approved in Brazil in December 2014 for the treatment of adult patients with moderately to severely active RA who have had an inadequate response to 1 or more DMARDs, and tofacitinib may be used in combination with csDMARDs or as monotherapy.^[24]^ Recently, an SBR position paper recommended that tofacitinib as monotherapy or in combination with MTX can be used as an alternative treatment for patients with RA with moderate or high disease activity after failure of at least 2 different csDMARDs and at least 1 bDMARD.^[25]^ Nevertheless, these recommendations stated that earlier use of tofacitinib may be considered under certain conditions, at the physician's discretion, based on evidence of the efficacy of tofacitinib at different times of treatment.

In order to expand the evidence base for the clinical use of tofacitinib as a treatment for RA in Brazil, we report the results of a pooled post-hoc analysis of efficacy and safety data from a cohort of Brazilian patients with RA who received tofacitinib 5 or 10 mg BID or placebo in global P2 and P3 studies.

## 2 Materials and methods

### 2.1 Patients

This post-hoc analysis included pooled efficacy and safety data from patients (aged ≥18 years) with active RA enrolled from Brazil in 3 global P2 studies [A3921019 (NCT00147498)^[11]^; A3921025 (NCT00413660)^[14]^; A3921035 (NCT00550446)^[13]^] and 3 global P3 studies of tofacitinib [ORAL Step, A3921032 (NCT00960440)^[18]^; ORAL Scan, A3921044 (NCT00847613)^[19]^; ORAL Solo, A3921045 (NCT00814307)^[17]^]. Full details of these studies, including patient inclusion and exclusion criteria, have been reported previously.^[11,13,14,17–19]^ Patients were required to have had an inadequate response to at least 1 csDMARD or bDMARD. Patients enrolled in the P2 studies had an inadequate response to MTX (A3921025); MTX or TNFi (A3921019); or csDMARDs (A3921035). Patients enrolled in the P3 studies had an inadequate response to MTX (ORAL Scan), csDMARDs or bDMARDs (ORAL Solo), or TNFi (ORAL Step). Key exclusion criteria for all studies included a significant infection within 6 months; a white blood cell count of < 3.0 x 10^3^/mm^3^; an absolute neutrophil count <1.2 × 10^3^/mm^3^; recurrent herpes zoster (HZ) or disseminated herpes simplex virus infections; infection with human immunodeficiency virus, hepatitis B or hepatitis C; or a history of, or existing, malignancy (other than adequately treated or excised nonmelanoma skin cancer or cervical carcinoma in situ).

In P2 studies, the presence of TB was determined by a positive Mantoux purified protein derivative (PPD) skin test or chest radiograph suggestive of active infection within the 3 months before randomization. In P3 studies, a negative screening for TB was defined by a negative QuantiFERON-Gold test or, if unavailable, a negative PPD skin test and a chest radiograph with no evidence of TB infection, both within the 3 months before randomization; and no prior history of either untreated or inadequately treated latent or active TB infection. In P2 studies, patients with positive TB screening were excluded. In P3 studies, patients with latent TB or inadequately treated latent TB were permitted entry into the studies following at least 1 month of a 9-month preventative regimen of isoniazid therapy.

### 2.2 Study design

The 3 P2 studies (A3921019,^[11]^ A3921025,^[14]^ and A3921035^[13]^) and 3 P3 studies (ORAL Step,^[18]^ ORAL Scan,^[19]^ and ORAL Solo^[17]^) included in this analysis were randomized, double-blind, placebo-controlled clinical trials. In P2 studies, which were of 6 weeks to 6 months in duration, patients were randomized to receive tofacitinib 1, 3, 5, 10, or 15 mg BID, tofacitinib 30 mg BID (A3921019), tofacitinib 20 mg once daily (QD; A3921025) or placebo, as monotherapy (A3921035 and A3921019) or in combination with background MTX (A3921025). Study A3921035 also included a monotherapy arm of adalimumab 40 mg administered subcutaneously once every 2 weeks. Patients in studies A3921025 and A3921035 who were classified as nonresponders (did not achieve ≥20% improvement in swollen and tender joint counts) at Month 3 were reassigned in a blinded manner to receive tofacitinib 5 mg BID.

Patients in P3 studies, which were of 6 to 24 months in duration, were randomized to receive tofacitinib 5 mg BID, tofacitinib 10 mg BID, or placebo with either background MTX (ORAL Step and ORAL Scan) or as monotherapy (ORAL Solo). Patients randomized to placebo in ORAL Step and ORAL Solo were advanced to tofacitinib 5 or 10 mg BID at Month 3, according to the sequence to which they were randomized at baseline. In ORAL Scan, patients randomized to placebo who were classified as nonresponders at Month 3 were advanced to tofacitinib 5 or 10 mg BID according to randomization at baseline; all remaining patients receiving placebo were advanced to tofacitinib 5 or 10 mg BID at Month 6. Only Brazilian patients who received treatment with tofacitinib 5 mg BID, tofacitinib 10 mg BID, or placebo were included in this analysis; MTX-naive patients were not included.

All studies were conducted in accordance with the Declaration of Helsinki, International Conference on Harmonisation Good Clinical Practice Guidelines, and were approved by the Institutional Review Board and/or Independent Ethics Committee of the investigational centers. All patients provided written, informed consent.

### 2.3 Efficacy analysis

Efficacy data for the cohort of Brazilian patients were pooled for analysis. The following efficacy outcomes were analyzed: American College of Rheumatology (ACR) 20/50/70 response rates; change from baseline in Disease Activity Score in 28 joints, erythrocyte sedimentation rate [DAS28-4(ESR)]; and change from baseline in Health Assessment Questionnaire-Disability Index (HAQ-DI). Efficacy was evaluated up to Month 24. Efficacy results were compared for both tofacitinib doses versus placebo at Month 3 (before placebo advancement to tofacitinib).

In addition to the HAQ-DI score, changes from baseline in the following patient-reported outcomes (PROs) were also evaluated: pain assessed utilizing a visual analog scale [pain (VAS)]; health-related quality of life (HRQoL) evaluated by the Medical Outcomes Study Short Form-36 Health Survey (SF-36) physical component summary (PCS) score and mental component summary (MCS) score; and fatigue assessed using the Functional Assessment of Chronic Illness Therapy-Fatigue (FACIT-F).

### 2.4 Safety analysis

Adverse events (AEs) were summarized for up to 24 months’ treatment.

### 2.5 Analyses

All efficacy and safety analyses were based on observed cases (i.e., no imputation) of the full analysis set (FAS), which included all patients who were randomized and received at least 1 dose of study treatment (tofacitinib or placebo). All analyses were descriptive in nature, with general trends described; statistical comparisons between treatment groups were not performed due to the small sample size.

## 3 Results

### 3.1 Patients

Of a global patient population of 2315 patients who received tofacitinib 5 or 10 mg BID or placebo in global P2 and P3 studies, a total of 226 patients from Brazil (9.8% of the global population) were randomized to treatment and were included in these analyses. Patients received tofacitinib 5 mg BID (96 of 878 patients were from Brazil; 10.9% of the global population); tofacitinib 10 mg BID (73 of 830 patients were from Brazil; 8.8% of the global population); or placebo (57 of 607 patients were from Brazil; 9.4% of the global population) either as monotherapy or in combination with MTX.

Overall, 190 (84.1%) patients completed the studies. Patient disposition is presented in Tables, Supplemental Digital Content 1 to 3, for P2 studies, P3 studies of 6 months’ duration, and P3 studies of 24 months’ duration, respectively. Reasons for premature withdrawal for patients in the Brazilian subpopulation are presented in Table 1.

**Table 1:**
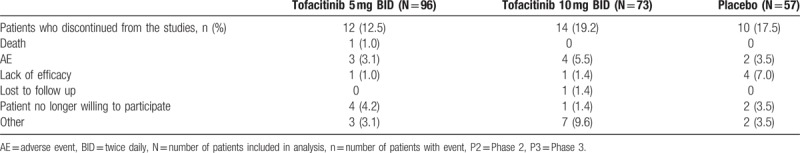
Patient discontinuations in the Brazilian P2/P3 subpopulation.

Demographics and baseline characteristics for the cohort of Brazilian patients are summarized in Table 2. In general, baseline demographics were similar across groups. The majority of patients were female (≥89.0%) and mean age ranged from 48.6 to 50.8 years. Baseline characteristics indicated patients were typically overweight (body mass index ≥25.0), had long-standing disease, and had a high level of baseline disease activity.

**Table 2:**
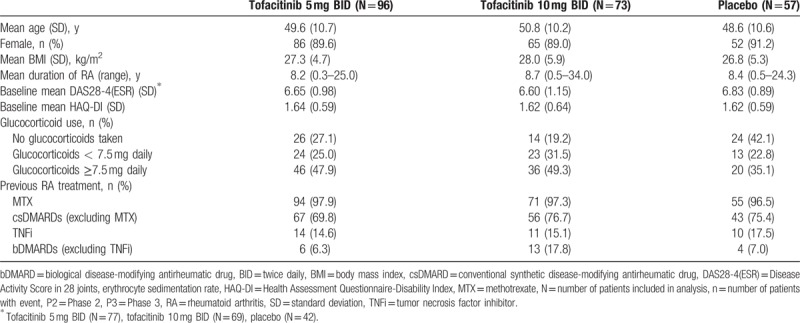
Baseline demographics and disease characteristics of Brazilian P2/P3 subpopulation.

### 3.2 Efficacy

#### 3.2.1 Disease signs and symptoms

**Figure 1:**
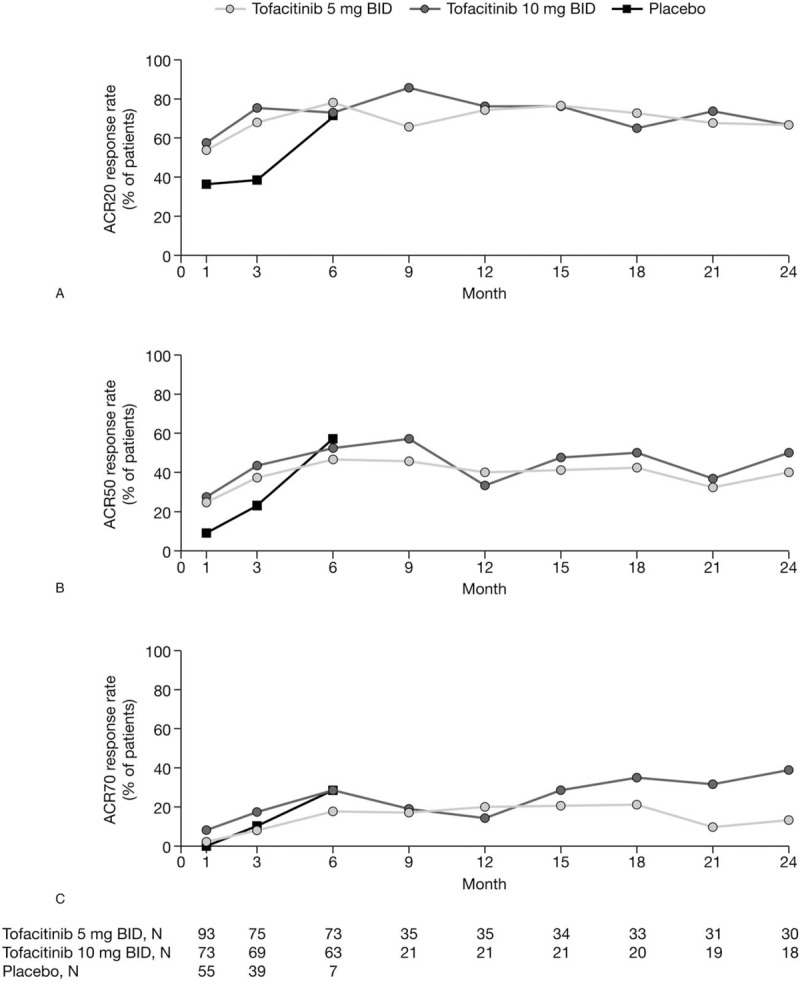
(A) ACR20 response rates, (B) ACR50 responses rates, and (C) ACR70 response rates over time (full analysis set, no imputation). Patients remaining in the placebo group up to Month 6 were those with at least 20% improvement in both tender/painful and swollen joint counts at Month 3 in ORAL Scan. ACR = American College of Rheumatology, BID = twice daily.

At Month 3, 68.0% (51/75) of patients treated with tofacitinib 5 mg BID and 75.4% (52/69) treated with tofacitinib 10 mg BID achieved ACR20 compared with 38.5% (15/39) of placebo-treated patients (Fig. 1A; data for Figure 1 are provided in Table, Supplemental Digital Content 4). Response rates in the tofacitinib 5 mg BID and tofacitinib 10 mg BID groups were generally sustained over 24 months. At Month 24, ACR20 response rates in the tofacitinib 5 mg BID and tofacitinib 10 mg BID groups were both 66.7% (Fig. 1A).

ACR50 response rates at Month 3 were 37.3% (28/75) with tofacitinib 5 mg BID, 43.5% (30/69) with tofacitinib 10 mg BID, and 23.1% (9/39) with placebo (Fig. 1B). At Month 24, the percentage of patients achieving an ACR50 response with tofacitinib 5 and 10 mg BID was 40.0% and 50.0%, respectively. At Month 3, 8.0% and 17.4% of patients treated with tofacitinib 5 mg BID and tofacitinib 10 mg BID, respectively, achieved ACR70 versus 10.3% of placebo-treated patients (Fig. 1C). At Month 24, ACR70 was achieved by 13.3% and 38.9% of patients treated with tofacitinib 5 and 10 mg BID, respectively.

**Figure 2:**
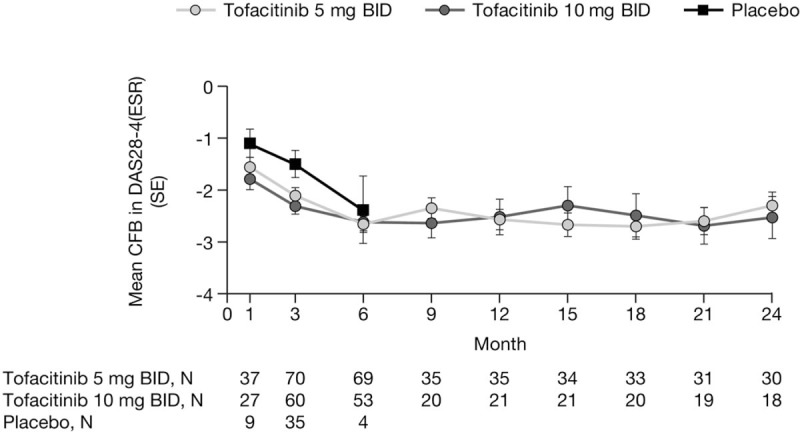
Mean change from baseline in DAS28-4(ESR) over time (efficacy population, full analysis set, no imputation). Patients remaining in the placebo group up to Month 6 were those with at least 20% improvement in both tender/painful and swollen joint counts at Month 3 in ORAL Scan. BID = twice daily, CFB = change from baseline, DAS28-4(ESR) = Disease Activity Score in 28 joints, erythrocyte sedimentation rate, SE = standard error.

At Month 3, the mean change from baseline in DAS28-4(ESR) was -2.11 with tofacitinib 5 mg BID, -2.31 with tofacitinib 10 mg BID, and -1.50 with placebo (Fig. 2; data for Figure 2 are provided in Table, Supplemental Digital Content 5). Thereafter, improvements from baseline were generally maintained up to Month 24 with tofacitinib treatment.

#### 3.2.2 Physical function and other patient-reported outcomes

**Figure 3:**
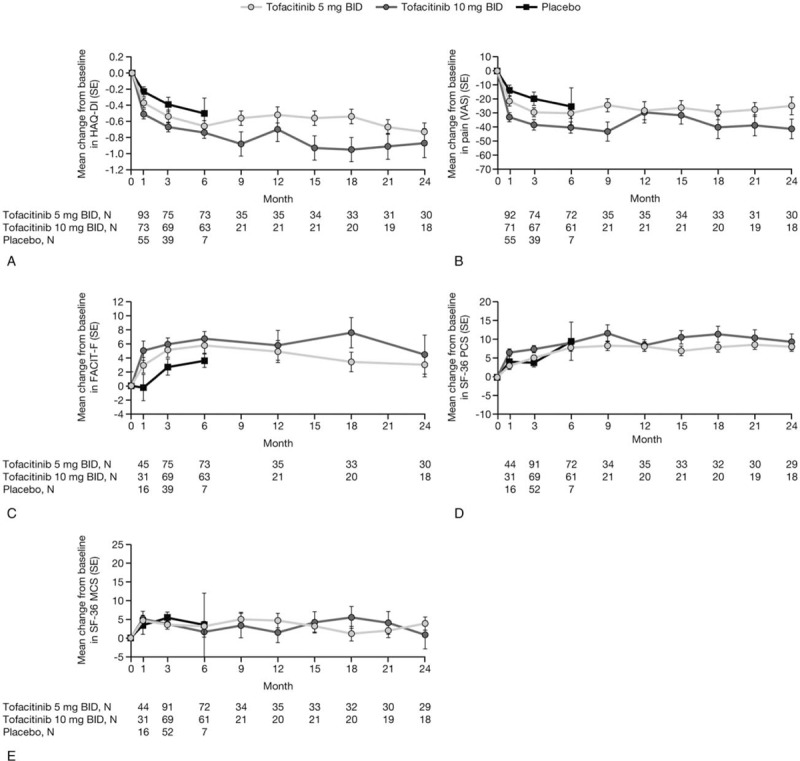
Mean changes from baseline in (A) HAQ-DI, (B) pain (VAS), (C) FACIT-F, (D) SF-36 PCS, and (E) SF-36 MCS (full analysis set, no imputation). Patients remaining in the placebo group up to Month 6 were those with at least 20% improvement in both tender/painful and swollen joint counts at Month 3 in ORAL Scan. BID = twice daily, FACIT-F = Functional Assessment of Chronic Illness Therapy-Fatigue, HAQ-DI = Health Assessment Questionnaire-Disability Index, MCS = mental component summary, pain = patient assessment of arthritis pain, PCS = physical component summary, SE = standard error, SF-36 = Short Form-36 Health Survey, VAS = visual analog scale.

At Month 3, mean change from baseline in HAQ-DI was -0.54 for patients treated with tofacitinib 5 mg BID, −0.67 for those treated with tofacitinib 10 mg BID, and −0.39 for those treated with placebo (Fig. 3A; data for Figure 3 are provided in Table, Supplemental Digital Content 6). Over 24 months of treatment, analysis of HAQ-DI and other PROs showed improvements from baseline with both tofacitinib doses in patient's assessment of arthritis pain (reduction from baseline; Fig. 3B); fatigue (FACIT-F, increase from baseline; Fig. 3C); HRQoL in terms of SF-36 PCS (increase from baseline; Fig. 3D); and SF-36 MCS (increase from baseline; Fig. 3E).

### 3.3 Safety

During the first 3 months of treatment (before patients receiving placebo advancing to tofacitinib), the incidence of treatment-emergent AEs (TEAEs) was similar between tofacitinib- and placebo-treated patients (Table 3). During Months 3 to 6, 60.2% of patients receiving tofacitinib 5 mg BID and 54.4% receiving tofacitinib 10 mg BID had TEAEs.

**Table 3:**
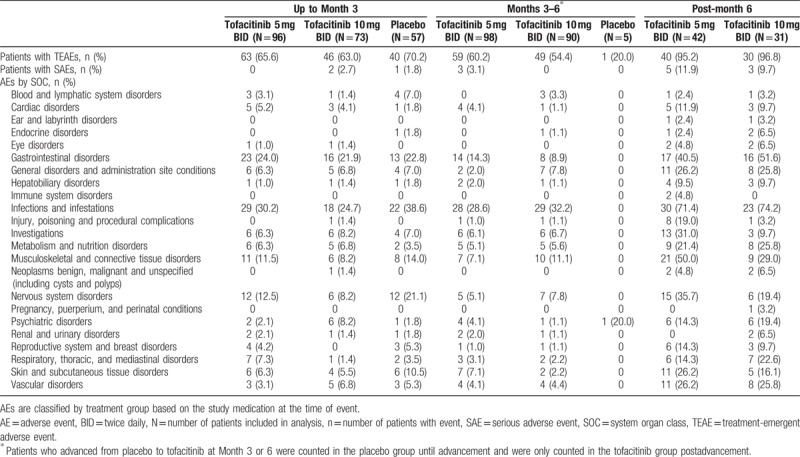
Summary of treatment-emergent AEs and SAEs by treatment group and treatment period.

Post-month 6, the percentage of patients treated with tofacitinib 5 mg BID and tofacitinib 10 mg BID with TEAEs was 95.2% and 96.8%, respectively. In general, across treatment groups, the most frequently reported TEAEs by system organ class were infections and infestations, gastrointestinal disorders, musculoskeletal and connective tissue disorders, and nervous system disorders (Table 3). Seven patients discontinued treatment due to AEs while receiving tofacitinib (3 and 4 patients in the tofacitinib 5 mg BID and tofacitinib 10 mg BID groups, respectively). Two patients in the placebo group discontinued treatment due to AEs. A summary of most frequent (occurring in ≥10% in any treatment group during each time period) TEAEs by PT and by treatment group (safety population) is provided in Table, Supplemental Digital Content 7.

A total of 13 tofacitinib-treated patients reported 17 SAEs, and 1 placebo-treated patient reported 1 SAE during the studies (Table 3). SAEs were related to the study drug (investigator-determined) in 8 patients. These included 4 events in 1 patient receiving tofacitinib 10 mg BID (drug interaction, lack of effect of oral contraceptive, unintended pregnancy, and missed abortion), 2 events in 1 patient receiving tofacitinib 5 mg BID (cardiac arrest and respiratory arrest), and 1 event each in 6 patients (tofacitinib 10 mg BID: pneumonia, liver abscess, and bacterial arthritis; tofacitinib 5 mg BID: bronchopneumonia, atypical pneumonia, and right bundle branch block).

AEs of special interest are reported in Table 4. There were 8 patients with SAEs in the tofacitinib 5 mg BID treatment group, 5 in the tofacitinib 10 mg BID treatment group, and 1 in the placebo group. There were 3 patients with serious infections in each tofacitinib treatment group and none in the placebo group (serious infections are included in the reporting of SAEs above). Serious infections in the tofacitinib 5 mg BID group were bronchopneumonia in a female patient aged 66 years who continued with tofacitinib treatment and recovered; atypical pneumonia in a female patient aged 62 years who permanently discontinued tofacitinib treatment and recovered; and pneumonia in a female patient aged 61 years who permanently discontinued tofacitinib treatment and recovered. In the tofacitinib 10 mg BID group, serious infections were liver abscess in a male patient aged 51 years who permanently discontinued treatment and recovered; pneumonia in a female patient aged 47 years who temporarily discontinued tofacitinib treatment and recovered; and bacterial arthritis in a female patient aged 28 years who permanently discontinued tofacitinib treatment and was reported as recovering.

**Table 4:**
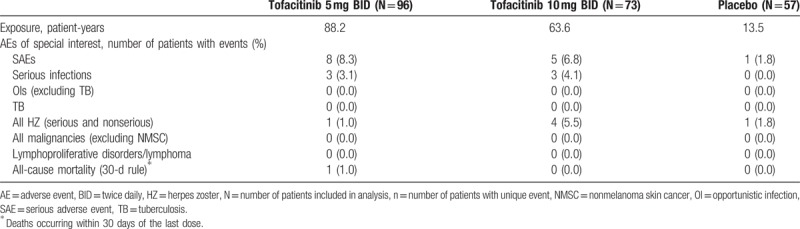
AEs of special interest by treatment group.

One patient treated with tofacitinib 5 mg BID, 4 patients treated with tofacitinib 10 mg BID, and 1 placebo-treated patient had HZ infection; no cases of serious HZ infection occurred. No cases of OI, lymphoproliferative disorders/lymphoma, TB, or malignancies [excluding nonmelanoma skin cancer (NMSC)] were reported in any treatment group, nor were there any occurrences of endemic fungal infections.

One death (within 30 days of last study drug dose) was reported in the tofacitinib 5 mg BID treatment group (Table 4). The patient was a female aged 57 years who experienced cardiac arrest and respiratory arrest on Day 611 of the study. The investigator considered the SAEs of cardiac and respiratory arrest leading to death as possibly related to study drug.

## 4 Discussion

In this analysis of the Brazilian subpopulation of treatment-refractory patients with RA pooled across P2 and P3 studies, tofacitinib 5 mg BID and tofacitinib 10 mg BID, as monotherapy or in combination with MTX, demonstrated efficacy in reducing signs and symptoms of RA, and improving physical function. Most patients in the Brazilian subpopulation were female, had a mean disease duration of 8.2 to 8.7 years, and had considerable disease activity, which was generally consistent with the patient demographics and disease characteristics in the overall populations in the P2 and P3 studies evaluated.^[11,13,14,17–19]^

Across the P2/P3 studies, improvements in ACR response rates and DAS28-4(ESR) and HAQ-DI scores were observed with tofacitinib treatment at Month 3. Consistent with these results, treatment with tofacitinib also resulted in improvements from baseline across multiple PROs (i.e., pain, fatigue, HRQoL). Improvements in efficacy outcomes were sustained for up to 24 months of treatment with tofacitinib; however, it should be noted that data to Month 24 were obtained from a single study (ORAL Scan) and included a small number of patients. Accordingly, these data should be interpreted with caution. In general, the efficacy profile of tofacitinib observed in Brazilian patients was similar to that reported previously for the global RA population^[11,13,14,17–19]^ and with the Latin American subpopulation of global P2 and P3 studies.^[26]^

In the pooled Brazilian P2/P3 RA population, the overall safety profile of tofacitinib was consistent with findings from tofacitinib global studies of up to 24 months’ duration^[11,13,14,17–19]^ and with the Latin American subpopulation of global studies.^[26,27]^ The most commonly reported AEs in the pooled P2/P3 Brazilian population with RA were infections and infestations. It is known that patients with RA are at an increased risk for infection, including TB and other opportunistic infections (OIs), compared with the general population,^[28]^ and this risk is further increased in patients treated with immunosuppressive drugs.^[29,30]^

Moreover, the risk of TB varies according to the background TB rate in the underlying population.^[31]^ No cases of TB were reported in patients with RA from Brazil treated with tofacitinib, despite the high incidence of TB in Brazil (41 cases per 100,000 people and 20th highest absolute number of cases globally).^[32]^ These results were consistent with the incidence rate (IR) for TB observed in the Latin American and rest of the world (RoW) subpopulation of global tofacitinib studies [IR, patients with events per 100 patient-years (95% confidence interval; CI) 0.05 (0.01–0.33) and 0.24 (0.16–0.35), respectively].^[27]^ It must be noted that in the tofacitinib clinical development program, patients were screened for latent or untreated TB, and it has been suggested that screening and treating latent TB infections should be employed before initiating tofacitinib treatment,^[33]^ similar to existing SBR recommendations for bDMARD therapy.^[5]^

For OIs other than TB, no events were reported in the Brazilian population with RA. In comparison, for the Latin American and RoW subpopulations of global tofacitinib studies, IRs (95% CI) for OIs were 0.28 (0.13–0.62) and 0.25 (0.17–0.36), respectively.^[27]^ OIs have also been reported with bDMARDs, and a recent meta-analysis has shown that patients receiving bDMARDs are 79% more likely to develop OIs than placebo-treated patients (odds ratio 1.79; 95% CI 1.17–2.74).^[34]^ However, heterogeneity in methodology and differences in the definitions for OIs make it difficult to directly compare rates of OIs between different RA treatments and studies.

In global tofacitinib studies, increased rates of HZ were observed in patients treated with tofacitinib compared with those receiving placebo, particularly among patients from Japan and Korea.^[35]^ In our pooled analysis of the Brazilian P2/P3 tofacitinib RA subpopulation, HZ cases were reported in both tofacitinib treatment groups, although none were serious. Data from the Brazilian biologic registry, BiobadaBrasil, have shown that the rate of HZ infection is increased with bDMARDs compared with csDMARDs, with a reported incidence of 4.8% versus 0%, respectively.^[36]^ As HZ is a preventable disease, the SBR recommends vaccination against HZ in patients ≥50 years before the use of csDMARDs or bDMARDs.^[37]^ A study of live zoster vaccine in patients with RA demonstrated that patients who were vaccinated 2 to 3 weeks before initiating tofacitinib treatment had similar humoral and cell-mediated immune responses to the vaccine compared with vaccinated patients who received placebo.^[38]^

Certain types of malignancies are more prevalent in patients with RA than in the general population.^[39,40]^ The mechanisms underlying this observation are not fully understood, but the immune response and some RA treatments can affect malignancy rates.^[39,41,42]^ No cases of malignancy (excluding NMSC) or lymphoma were reported with tofacitinib in our analysis of the pooled Brazilian P2/P3 RA population. However, it should be noted that this analysis was based on data from relatively short duration randomized clinical trials and malignancies typically only develop over a long latency period. IRs (95% CI) for malignancy (excluding NMSC) and lymphoma reported in the global tofacitinib program were 0.85 (0.70–1.02) and 0.08 (0.04–0.14), respectively,^[43]^ and IRs (95% CI) for malignancies (excluding NMSC) in the Latin American and RoW subpopulations of global tofacitinib studies were 0.42 (0.22–0.81) and 0.93 (0.77–1.14), respectively.^[27]^ These IRs for malignancy (excluding NMSC) were consistent with those reported in the CORRONA International registry of patients with RA (0.61 events per 100 patient-years), which included data from Eastern Europe, Latin America, and India.^[44]^

The comparatively low number of patients and relatively short duration of observation in our analysis may limit the conclusions that can be drawn. It is therefore important to carefully monitor the safety of tofacitinib, as already recommended by the SBR and other rheumatology societies in Latin America for the use of therapeutic agents in the treatment of RA.^[5]^ Due to the small sample sizes, no formal statistical analyses were conducted to directly compare the efficacy and safety of tofacitinib with placebo. Other limitations included the use of post-hoc analyses and the pooling of data from studies with differing study designs and methodologies, resulting in a heterogeneous patient population.

Current challenges for the treatment of RA in Brazil, and other Latin American countries, include increasing access to prompt diagnosis, treatment by rheumatologists, and the availability of appropriate therapy.^[3,45,46]^ Despite the availability of bDMARDs in Brazil, evidence suggests that few patients (<10% of those with moderate to severe RA) are prescribed these treatments.^[47]^ This may be a consequence of poor access to treatment and attitudinal factors (e.g., fear of needles). Therefore, orally administered treatments such as tofacitinib may be advantageous and offer benefits in rural areas where infrastructure/experience with parenteral administration and patient access to clinics are limited and may consequently reduce costs of treatment. Moreover, tofacitinib, with its different mechanism of action to existing DMARDs, may provide an alternative treatment option for patients with an inadequate response to other therapies. Indeed, the results of our analyses demonstrated the effectiveness of tofacitinib, as monotherapy or in combination with MTX, in Brazilian patients with RA who had an inadequate response to prior csDMARD or bDMARD therapy. Tofacitinib is included in recent SBR recommendations for patients who have failed at least 2 different csDMARDs and at least 1 bDMARD and this analysis provides evidence to support tofacitinib as an additional agent in the current armamentarium of treatments for RA in Brazil. Current ACR and EULAR guidelines recommend tofacitinib treatment for patients with RA who do not adequately respond to therapy with csDMARDs.^[48,49]^ Continued evaluation of data from long-term extension studies of tofacitinib will be important in assessing its sustained efficacy and tolerability in the Brazilian subpopulation.^[22,23]^ Reports for Latin American countries are very important in providing a continental evaluation of this new treatment.

In conclusion, treatment with tofacitinib 5 mg BID and tofacitinib 10 mg BID resulted in improvements in disease signs and symptoms, and improved physical function of up to 24 months in Brazilian patients from P2 and P3 studies. The safety profile of tofacitinib in the Brazilian subpopulation was consistent with that of both LA and global populations up to 24 months.

## Author contributions

**Conceptualization:** Sebastião C. Radominski, Flora Marcolino, Claiton V. Brenol, Cristiano A.F. Zerbini, Erika G. García, Ermeg L. Akylbekova, Ricardo Rojo, Dario Ponce de Leon.

**Investigation:** Andrea B.V. Lomonte, Sebastião C. Radominski, Flora Marcolino, Claiton V. Brenol, Cristiano A.F. Zerbini, Erika G. García, Ermeg L. Akylbekova, Ricardo Rojo, Dario Ponce de Leon.

**Supervision:** Andrea B.V. Lomonte, Sebastião C. Radominski, Flora Marcolino, Claiton V. Brenol, Cristiano A.F. Zerbini, Erika G. García, Ermeg L. Akylbekova, Ricardo Rojo, Dario Ponce de Leon.

**Validation:** Andrea B.V. Lomonte, Sebastião C. Radominski, Flora Marcolino, Claiton V. Brenol, Cristiano A.F. Zerbini, Erika G. García, Ermeg L. Akylbekova, Ricardo Rojo, Dario Ponce de Leon.

**Visualization:** Andrea B.V. Lomonte, Sebastião C. Radominski, Flora Marcolino, Claiton V. Brenol, Cristiano A.F. Zerbini, Erika G. García, Ermeg L. Akylbekova, Ricardo Rojo, Dario Ponce de Leon.

**Writing – original draft:** Andrea B.V. Lomonte, Sebastião C. Radominski, Flora Marcolino, Claiton V. Brenol, Cristiano A.F. Zerbini, Erika G. García, Ermeg L. Akylbekova, Ricardo Rojo, Dario Ponce de Leon.

**Writing – review & editing:** Andrea B.V. Lomonte, Sebastião C. Radominski, Flora Marcolino, Claiton V. Brenol, Cristiano A.F. Zerbini, Erika G. García, Ermeg L. Akylbekova, Ricardo Rojo, Dario Ponce de Leon.

## Supplementary Material

Supplemental Digital Content
