# Supplementary material for: Tofacitinib, an oral Janus kinase inhibitor, in patients from Brazil with rheumatoid arthritis: Pooled efficacy and safety analyses
Source: Medicine (Baltimore). 2018 Aug 3;97(31):e11609. doi: 10.1097/MD.0000000000011609 (PMC6081087; doi:10.1097/MD.0000000000011609)
Supplement: Supplemental Digital Content [file medi-97-e11609-s001.docx]

**Supplemental Digital Content 1.** Patient Disposition for Phase 2 Studies.

|  | **Tofacitinib 5 mg BID (N = 21)** | **Tofacitinib 10 mg BID (N = 8)** | **Placebo (N = 19)** | **Placebo advanced to tofacitinib 5 mg BID (N = 1)** |
| --- | --- | --- | --- | --- |
| **Visit** | **n (%)** | **n (%)** | **n (%)** | **n (%)** |
| Baseline | 3 (14.3) | 8 (100.0) | 4 (21.1) | 1 (100.0) |
| Month 1 | 20 (95.2) | 8 (100.0) | 18 (94.7) | 1 (100.0) |
| Month 2 | 21 (100.0) | 8 (100.0) | 18 (94.7) | 1 (100.0) |
| Month 3 | 3 (14.3) | 7 (87.5) | 3 (15.8) | 1 (100.0) |
| Month 6 | 3 (14.3) | 7 (87.5) | 3 (15.8) | 1 (100.0) |

Patient disposition is based on presence of observed ACR20 endpoint in the FAS.

ACR20, American College of Rheumatology 20% response; BID twice daily; FAS, full analysis set.

**Supplemental Digital Content 2.** Patient Disposition for Phase 3 Studies of 6 Months’ Duration.

|  | **Tofacitinib 5 mg BID (N = 37)** | **Tofacitinib 10 mg BID (N = 39)** | **Placebo advanced to tofacitinib 5 mg BID (N = 15)** | **Placebo advanced to tofacitinib 10 mg BID (N = 13)** |
| --- | --- | --- | --- | --- |
| **Visit** | **n (%)** | **n (%)** | **n (%)** | **n (%)** |
| Baseline | 37 (100.0) | 39 (100.0) | 15 (100.0) | 13 (100.0) |
| Month 1 | 36 (97.3) | 39 (100.0) | 15 (100.0) | 12 (92.3) |
| Month 2 | 28 (75.7) | 34 (87.2) | 10 (66.7) | 10 (76.9) |
| Month 3 | 35 (94.6) | 37 (94.9) | 15 (100.0) | 11 (84.6) |
| Month 6 | 34 (91.9) | 34 (87.2) | 14 (93.3) | 9 (69.2) |

Patient disposition is based on presence of observed ACR20 endpoint in the FAS.

ACR20, American College of Rheumatology 20% response; BID twice daily; FAS, full analysis set.

**Supplemental Digital Content 3.** Patient Disposition for Phase 3 Study of 24 Months’ Duration.

|  | **Tofacitinib 5 mg BID (N = 38)** | **Tofacitinib 10 mg BID (N = 26)** | **Placebo advanced to tofacitinib 5 mg BID (N = 4)** | **Placebo advanced to tofacitinib 10 mg BID (N = 5)** |
| --- | --- | --- | --- | --- |
| **Visit** | **n (%)** | **n (%)** | **n (%)** | **n (%)** |
| Baseline | 38 (100.0) | 26 (100.0) | 4 (100.0) | 5 (100.0) |
| Month 1 | 37 (97.4) | 26 (100.0) | 4 (100.0) | 5 (100.0) |
| Month 3 | 37 (97.4) | 25 (96.2) | 4 (100.0) | 5 (100.0) |
| Month 6 | 36 (94.7) | 22 (84.6) | 4 (100.0) | 4 (80.0) |
| Month 9 | 35 (92.1) | 21 (80.8) | 4 (100.0) | 4 (80.0) |
| Month 12 | 35 (92.1) | 21 (80.8) | 4 (100.0) | 4 (80.0) |
| Month 15 | 34 (89.5) | 21 (80.8) | 4 (100.0) | 4 (80.0) |
| Month 18 | 33 (86.8) | 20 (76.9) | 4 (100.0) | 4 (80.0) |
| Month 21 | 31 (81.6) | 19 (73.1) | 4 (100.0) | 4 (80.0) |
| Month 24 | 30 (79.0) | 18 (69.2) | 4 (100.0) | 4 (80.0) |

Patient disposition is based on presence of observed ACR20 endpoint in the FAS.

ACR20, American College of Rheumatology 20% response; BID twice daily; FAS, full analysis set.

**Supplemental Digital Content 4.** Data for Figure 1. ACR20 Response Rates, ACR50 Responses Rates, and ACR70 Response Rates over Time (Efficacy Population, Full Analysis Set, No Imputation).

| **Month** | **Tofacitinib 5 mg BID** | | **Tofacitinib 10 mg BID** | | **Placebo** | |
| --- | --- | --- | --- | --- | --- | --- |
|  | **N** | **ACR response, %** | **N** | **ACR response, %** | **N** | **ACR response, %** |
| **ACR20** | | | | | | |
| 1 | 93 | 53.8 | 73 | 57.5 | 55 | 36.4 |
| 3 | 75 | 68.0 | 69 | 75.4 | 39 | 38.5 |
| 6 | 73 | 78.1 | 63 | 73.0 | 7 | 71.4 |
| 9 | 35 | 65.7 | 21 | 85.7 | - | - |
| 12 | 35 | 74.3 | 21 | 76.2 | - | - |
| 15 | 34 | 76.5 | 21 | 76.2 | - | - |
| 18 | 33 | 72.7 | 20 | 65.0 | - | - |
| 21 | 31 | 67.7 | 19 | 73.7 | - | - |
| 24 | 30 | 66.7 | 18 | 66.7 | - | - |
| **ACR50** | | | | | | |
| 1 | 93 | 24.7 | 73 | 27.4 | 55 | 9.1 |
| 3 | 75 | 37.3 | 69 | 43.5 | 39 | 23.1 |
| 6 | 73 | 46.6 | 63 | 52.4 | 7 | 57.1 |
| 9 | 35 | 45.7 | 21 | 57.1 | - | - |
| 12 | 35 | 40.0 | 21 | 33.3 | - | - |
| 15 | 34 | 41.2 | 21 | 47.6 | - | - |
| 18 | 33 | 42.4 | 20 | 50.0 | - | - |
| 21 | 31 | 32.3 | 19 | 36.8 | - | - |
| 24 | 30 | 40.0 | 18 | 50.0 | - | - |
| **ACR 70** | | | | | | |
| 1 | 93 | 2.2 | 73 | 8.2 | 55 | 0 |
| 3 | 75 | 8.0 | 69 | 17.4 | 39 | 10.3 |
| 6 | 73 | 17.8 | 63 | 28.6 | 7 | 28.6 |
| 9 | 35 | 17.1 | 21 | 19.0 | - | - |
| 12 | 35 | 20.0 | 21 | 14.3 | - | - |
| 15 | 34 | 20.6 | 21 | 28.6 | - | - |
| 18 | 33 | 21.2 | 20 | 35.0 | - | - |
| 21 | 31 | 9.7 | 19 | 31.6 | - | - |
| 24 | 30 | 13.3 | 18 | 38.9 | - | - |

Patients remaining in the placebo group up to Month 6 were those with at least 20% improvement in both tender/painful and swollen joint counts at Month 3 in ORAL Scan.

ACR 20/50/70, American College of Rheumatology 20/50/70 response rates; BID, twice daily; N, number of patients included in analysis.

**Supplemental Digital Content 5.** Data for Figure 2. Mean Change from Baseline in DAS28-4(ESR) over Time (Efficacy Population, Full Analysis Set, No Imputation).

| **Month** | **Tofacitinib 5 mg BID** | | **Tofacitinib 10 mg BID** | | **Placebo** | |
| --- | --- | --- | --- | --- | --- | --- |
|  | **N** | **Mean CFB in DAS28-4(ESR) (SE)** | **N** | **Mean CFB in DAS28-4(ESR) (SE)** | **N** | **Mean CFB in DAS28-4(ESR) (SE)** |
| 1 | 37 | -1.56 (0.18) | 27 | -1.79 (0.20) | 9 | -1.10 (0.26) |
| 3 | 70 | -2.11 (0.15) | 60 | -2.31 (0.15) | 35 | -1.50 (0.25) |
| 6 | 69 | -2.66 (0.15) | 53 | -2.62 (0.16) | 4 | -2.39 (0.65) |
| 9 | 35 | -2.35 (0.19) | 20 | -2.64 (0.27) | - | - |
| 12 | 35 | -2.57 (0.19) | 21 | -2.52 (0.33) | - | - |
| 15 | 34 | -2.67 (0.22) | 21 | -2.30 (0.35) | - | - |
| 18 | 33 | -2.70 (0.24) | 20 | -2.49 (0.40) | - | - |
| 21 | 31 | -2.60 (0.25) | 19 | -2.69 (0.34) | - | - |
| 24 | 30 | -2.30 (0.25) | 18 | -2.53 (0.39) | - | - |

Patients remaining in the placebo group up to Month 6 were those with at least 20% improvement in both tender/painful and swollen joint counts at Month 3 in ORAL Scan.

BID, twice daily; CFB, change from baseline; DAS28-4(ESR), Disease Activity Score in 28 joints, erythrocyte sedimentation rate; N, number of patients included in analysis; SE, standard error.

**Supplemental Digital Content 6.** Data for Figure 3. Mean Changes from Baseline in HAQ-DI, pain, FACIT-F, SF-36 PCS, and SF-36 MCS (efficacy population, full analysis set, no imputation).

| **Month** | **Tofacitinib 5 mg BID** | | **Tofacitinib 10 mg BID** | | **Placebo** | |
| --- | --- | --- | --- | --- | --- | --- |
|  | **N** | **Mean CFB (SE)** | **N** | **Mean CFB (SE)** | **N** | **Mean CFB (SE)** |
| **HAQ-DI** | | | | | | |
| 1 | 93 | -0.37 (0.06) | 73 | -0.51 (0.06) | 55 | -0.23 (0.06) |
| 3 | 75 | -0.54 (0.08) | 69 | -0.67 (0.06) | 39 | -0.39 (0.09) |
| 6 | 73 | -0.66 (0.07) | 63 | -0.74 (0.07) | 7 | -0.50 (0.19) |
| 9 | 35 | -0.56 (0.09) | 21 | -0.88 (0.15) | - | - |
| 12 | 35 | -0.52 (0.10) | 21 | -0.70 (0.15) | - | - |
| 15 | 34 | -0.56 (0.09) | 21 | -0.93 (0.15) | - | - |
| 18 | 33 | -0.54 (0.09) | 20 | -0.95 (0.15) | - | - |
| 21 | 31 | -0.67 (0.09) | 19 | -0.91 (0.16) | - | - |
| 24 | 30 | -0.73 (0.11) | 18 | -0.87 (0.18) | - | - |
| **pain** | | | | | | |
| 1 | 92 | -21.4 (3.4) | 71 | -32.8 (3.2) | 55 | -13.7 (3.8) |
| 3 | 74 | -29.3 (3.2) | 67 | -38.2 (3.7) | 39 | -19.6 (4.8) |
| 6 | 72 | -29.9 (3.5) | 61 | -40.1 (4.0) | 7 | -25.4 (13.3) |
| 9 | 35 | -24.3 (4.7) | 21 | -43.0 (6.8) | - | - |
| 12 | 35 | -28.1 (6.5) | 21 | -29.3 (7.5) | - | - |
| 15 | 34 | -26.1 (5.2) | 21 | -31.4 (6.2) | - | - |
| 18 | 33 | -29.3 (5.3) | 20 | -40.0 (8.0) | - | - |
| 21 | 31 | -27.3 (4.9) | 19 | -38.5 (7.4) | - | - |
| 24 | 30 | -24.7 (6.3) | 18 | -41.1 (6.9) | - | - |
| **FACIT-F** | | | | | | |
| 1 | 45 | 2.9 (1.1) | 31 | 5.0 (1.4) | 16 | -0.3 (1.9) |
| 3 | 75 | 5.1 (1.0) | 69 | 5.9 (0.9) | 39 | 2.7 (1.1) |
| 6 | 73 | 5.7 (1.1) | 63 | 6.7 (1.0) | 7 | 3.6 (1.0) |
| 12 | 35 | 4.9 (1.6) | 21 | 5.8 (2.1) | - | - |
| 18 | 33 | 3.4 (1.4) | 20 | 7.6 (2.2) | - | - |
| 24 | 30 | 3.0 (1.7) | 18 | 4.4 (2.8) | - | - |
| **SF-36 PCS** | | | | | | |
| 1 | 44 | 3.1 (1.0) | 31 | 6.6 (1.0) | 16 | 4.1 (1.8) |
| 3 | 91 | 5.2 (0.8) | 69 | 7.5 (1.0) | 52 | 3.9 (1.1) |
| 6 | 72 | 7.9 (1.0) | 61 | 9.2 (1.2) | 7 | 9.4 (5.1) |
| 9 | 34 | 8.4 (1.3) | 21 | 11.7 (2.2) | - | - |
| 12 | 35 | 8.2 (1.3) | 20 | 8.5 (1.5) | - | - |
| 15 | 33 | 7.0 (1.3) | 21 | 10.6 (1.8) | - | - |
| 18 | 32 | 8.0 (1.3) | 20 | 11.4 (2.1) | - | - |
| 21 | 30 | 8.6 (1.2) | 19 | 10.5 (2.2) | - | - |
| 24 | 29 | 8.1 (1.2) | 18 | 9.4 (2.1) | - | - |
| **SF-36 MCS** | | | | | | |
| 1 | 44 | 4.6 (1.4) | 31 | 5.0 (2.0) | 16 | 3.3 (2.4) |
| 3 | 91 | 3.5 (1.2) | 69 | 3.7 (1.4) | 52 | 5.5 (1.4) |
| 6 | 72 | 3.1 (1.3) | 61 | 1.6 (1.4) | 7 | 3.5 (8.6) |
| 9 | 34 | 5.0 (1.8) | 21 | 3.3 (3.2) | - | - |
| 12 | 35 | 4.6 (1.9) | 20 | 1.5 (2.7) | - | - |
| 15 | 33 | 3.1 (1.6) | 21 | 4.1 (2.8) | - | - |
| 18 | 32 | 1.1 (1.9) | 20 | 5.4 (2.9) | - | - |
| 21 | 30 | 2.0 (1.9) | 19 | 4.0 (3.0) | - | - |
| 24 | 29 | 3.8 (1.8) | 18 | 0.8 (3.7) | - | - |

Patients remaining in the placebo group up to Month 6 were those with at least 20% improvement in both tender/painful and swollen joint counts at Month 3 in ORAL Scan.

BID, twice daily; CFB, change from baseline; FACIT-F, Functional Assessment of Chronic Illness Therapy-Fatigue; HAQ-DI, Health Assessment Questionnaire-Disability Index; MCS, mental component summary; N, number of patients included in analysis; pain, patient assessment of arthritis pain; PCS, physical component summary; SE, standard error; SF-36, Short Form-36 Health Survey.

**Supplemental Digital Content 7.** Summary of Most Frequent (Occurring in ≥10% in Any Treatment Group during Each Time Period) Treatment-emergent AEs by PT and by Treatment Group (Safety Population).

|  | **Up to Month 3** | | | **Month 3-6^a^** | | | **Post-month 6** | |
| --- | --- | --- | --- | --- | --- | --- | --- | --- |
|  | **Tofacitinib 5 mg BID**  **(N = 96)** | **Tofacitinib 10 mg BID**  **(N = 73)** | **Placebo**  **(N = 57)** | **Tofacitinib 5 mg BID**  **(N = 98)** | **Tofacitinib 10 mg BID**  **(N = 90)** | **Placebo**  **(N = 5)** | **Tofacitinib 5 mg BID**  **(N = 42)** | **Tofacitinib 10 mg BID**  **(N = 31)** |
| AEs by PT, n (%) | | | | | | | | |
| Upper respiratory tract infection | 5 (5.2) | 2 (2.7) | 4 (7.0) | 6 (6.1) | 2 (2.2) | 0 | 10 (23.8) | 8 (25.8) |
| Weight increased | 3 (3.1) | 4 (5.5) | 0 | 6 (6.1) | 2 (2.2) | 0 | 10 (23.8) | 2 (6.5) |
| Back pain | 5 (5.2) | 2 (2.7) | 1 (1.8) | 3 (3.1) | 5 (5.6) | 0 | 9 (21.4) | 4 (12.9) |
| Urinary tract infection | 3 (3.1) | 1 (1.4) | 2 (3.5) | 3 (3.1) | 4 (4.4) | 0 | 9 (21.4) | 5 (16.1) |
| Headache | 8 (8.3) | 3 (4.1) | 8 (14.0) | 3 (3.1) | 2 (2.2) | 0 | 8 (19.0) | 1 (3.2) |
| Nausea | 4 (4.2) | 4 (5.5) | 1 (1.8) | 4 (4.1) | 1 (1.1) | 0 | 8 (19.0) | 4 (12.9) |
| Hypertension | 1 (1.0) | 4 (5.5) | 2 (3.5) | 3 (3.1) | 3 (3.3) | 0 | 7 (16.7) | 6 (19.4) |
| Osteoarthritis | 0 | 0 | 0 | 0 | 0 | 0 | 6 (14.3) | 0 |
| Fall | 0 | 1 (1.4) | 0 | 0 | 0 | 0 | 5 (11.9) | 1 (3.2) |
| Hyper-cholesterolemia | 3 (3.1) | 3 (4.1) | 0 | 1 (1.0) | 1 (1.1) | 0 | 5 (11.9) | 3 (9.7) |
| Depression | 0 | 3 (4.1) | 0 | 1 (1.0) | 1 (1.1) | 0 | 3 (7.1) | 4 (12.9) |
| Dyspepsia | 5 (5.2) | 6 (8.2) | 2 (3.5) | 5 (5.1) | 2 (2.2) | 0 | 3 (7.1) | 5 (16.1) |
| Cystitis | 1 (1.0) | 0 | 1 (1.8) | 0 | 1 (1.1) | 0 | 2 (4.8) | 4 (12.9) |
| Dyslipidemia | 0 | 0 | 0 | 0 | 0 | 0 | 1 (2.4) | 4 (12.9) |
| Emotional disorder | 0 | 0 | 0 | 0 | 0 | 1 (20.0) | 0 | 0 |

AEs are classified into treatment groups based on the study medication at the time of event.

^a^Patients who advanced from placebo to tofacitinib at Month 3 or 6 were counted in the placebo group until advancement and were only counted in the tofacitinib group post-advancement.

AE, adverse event; BID, twice daily; N, number of patients included in analysis; n, number of patients with event; PT, preferred term.
